# Prognostic Significance of Central Pulse Pressure for Mortality in Patients With Coronary Artery Disease Receiving Repeated Percutaneous Coronary Intervention

**DOI:** 10.1097/MD.0000000000003218

**Published:** 2016-04-01

**Authors:** Mao-Jen Lin, Chun-Yu Chen, Hau-De Lin, Chung-Sheng Lin, Han-Ping Wu

**Affiliations:** From the Institue of Medicine(M-JL,C-SL),Chung-Shan Medical University, Taichung;Division of Cardiology (M-JL,H-DL), Department of Medicine, Taichung Tzu-Chi Hospital,the Buddhist Tzu-Chi Medical Foundation,Taichung; Department of Medicine (M-JL), School of Medicine, Tzu Chi University, Hualien; Division of Emergency Medicine (C-YC), Department of Pediatrics, Changhua Christian Hospital, Changhua; School of Medicine (C-YC), Kaohsiung Medical University, Kaohsiung; School of Medicine (C-YC, C-SL), Chung-Shan Medical University; Department of Internal Medicine (C-SL), Chung-Shan Medical University Hospital, Taichung; Division of Pediatric General Medicine (H-PW), Department of Pediatrics, Chang Gung Memorial Hospital at Linko, Kweishan; and College of Medicine (H-PW), Chang Gung University, Taoyuan, Taiwan, China.

## Abstract

Coronary artery disease (CAD) is a life-threatening medical emergency which needs urgent medical attention. Percutaneous coronary intervention (PCI) is common and necessary for patients with CAD, but it has not completely evaluated in cases with repeated PCI. Therefore, the aim of this study was to examine the risk factors and prognosis in patients with CAD requiring repeated PCI.

This is a prospective observational study. A total of 1126 patients with CAD requiring PCI took part in this study. Clinical parameters including baseline characteristics, hemodynamic data, location of vascular lesions, SYNTAX score, left ventricular ejection fraction, central pulse pressure (CPP), central aortic systolic pressure (CSP), risk factors, and invasive strategies were analyzed to identify the risk factors for patients requiring repeated PCI. We further analyzed the prognosis, including risk for myocardial infarction (MI), cardiovascular (CV) mortality, and all-cause mortality, in patients with repeated PCI.

Among patients with PCI, 276 received repeated PCI. Patients in the repeated PCI group had a higher CPP (66.7 vs 62.5 mm Hg; *P* = 0.006), CSP (139.9 vs 135.9 mm Hg; *P* = 0.017), and male preponderance (*P* = 0.012). Drugs including diuretics, beta-blockers (BBs), angiotensin-converting enzyme inhibitors (ACEIs), and aspirin were all used more frequently in the repeated PCI group (all *P* < 0.05). Freedom from MI was lower in the repeated PCI group than in the single PCI group (*P* < 0.001). Logistic regression revealed that CPP, CSP, number of diseased vessels, male sex, usage of diuretics, BBs, ACEIs, and MI were all predictors for requiring repeated PCI (all *P* < 0.05). In addition, CPP was a predictor for MI attack, CV mortality, and all-cause mortality in the repeated PCI group (*P* = 0.010, *P* = 0.041, *P* = 0.004, respectively).

Elevated CPP, CSP, male sex, multiple diseased vessels, and the usage of diuretics, BBs, ACEIs, and MI were predictors for repeated PCI. Most importantly, CPP was strongly associated with MI attack, CV mortality, and all-cause mortality, and could serve as a prognostic parameter for mortality in patients with CAD after performing repeated PCI.

## INTRODUCTION

Percutaneous coronary intervention (PCI) is common in patients with coronary artery disease (CAD). The term PCI refers to coronary revascularization through transarterial approach via a broad spectrum of the balloons, stents, and other devices. Athersclerotic plaque can be treated via different techniques such as expansion of the lumen by stretching and tearing the plaque (eg, balloon angioplasty), scaffolding the plaque (eg, coronary stents), removing the plaque (eg, atherectomy), or ablating the plaque (eg, laser agnioplasty). Repeated PCI may be indicated for some patients after first intervention. Even with the introduction of coronary stents, angiographic restenosis and clinical stenosis have still occurred in 20% to 30% and 10% to 15% of patients, respectively.^1,2^ The mechanism of restenosis results from one of the following mechanisms: negative remodeling after balloon angioplasty,^3^ intimal hyperplasia within the bare-metal stent; and local tissue growth within the drug-eluting stent or on its edge.^4^ Another cause of repeated procedures is the progression of atherosclerosis at a remote site, which is different from a previously treated lesion.^5^

Clinical outcomes of patients with PCI include myocardial infarction (MI), revascularization, and death.^6^ Indication for repeated revascularization may be associated with multiple vessel disease, presence of angina, and maximal stent length used.^7^ The revascularization techniques include redo-PCI and redo-coronary artery bypass graft (CABG). Prognosis of redo-CABG in patients with ischemic cardiomyopathy had been studied.^8^ However, analysis of clinical features and prediction of clinical outcome of patients with repeated PCI have not been well studied and clarified. Therefore, in this study, we aimed to survey the clinical features and outcome in patients with repeated PCI compared with those with single PCI, and further analyze the risk factors of MI and mortality in patients with repeated PCI.

## METHODS

### Study Population

This was a prospective observational study in design from 2007 to 2014. We consecutively recruited PCI patients aged between 20 and 95 years from the inpatient clinic in a medical center. Patients with CAD were divided into 2 groups: patients receiving a single PCI procedure and those who received repeated PCI procedures. In this study, PCI procedures included balloon angioplasty, bare-metal stent deployment, and drug-eluting stent deployment. In the repeated PCI group, patients with ischemia-driven clinical events for performing repeated PCI procedures were included. Patients with the following conditions—scheduled PCI and malignancy—were excluded. Most patients were followed up regularly via the outpatient department (OPD) basis. For few patients lost to follow-up at OPD, usually a telephone call would be used to contact the patients themselves or their families. For each patient, a survey on de novo MI, cardiovascular (CV) death, and all-cause death was completed at the end of the study. The Institution Review Board and ethics committee approved the study protocol and informed consent was obtained from all study participants.

### Data Collection, Measurements, and Analysis

Comparisons of body habitus, baseline biochemical data, homodynamic data (central aortic pressure [CAP], central aortic systolic pressure [CSP], central aortic diastolic pressure [CDP], central aortic mean pressure [CMP], central aortic pulse pressure [CPP], and central aortic systolic pressure [CSP]) on cardiac catheterization, exposed risk factors, and differences between treatment strategies such as drug medications or invasive procedures were all gathered in our study. The measurements of body parameters included body height, body weight, numbers of diseased vessel, location of lesions (left anterior descending artery [LAD], left circumflex artery [Lcx], and right coronary artery [RCA]), synergy between PCI with Taxus and Cardiac Surgery score (SYNTAX score), left ventricular ejection fraction (LVEF), and body mass index (BMI). Baseline biochemical data including fasting plasma glucose, creatinine, total cholesterol, high-density lipoprotein cholesterol (HDL-C), low-density lipoprotein cholesterol (LDL-C), and serum triglyceride level during first-time PCI were all collected. As for the hemodynamic data, CSP and CDP during first-time PCI were also collected. Diabetes was defined as a fasting plasma glucose level of more than 126 mg/dL, a casual plasma glucose level greater than 200 mg/dL, or a hemoglobin A1c (HbA1c) level of more than 6.5%.^9^ Hypercholesterolemia was defined as a serum cholesterol level of more than 200 mg/dL or an LDL-C level of more than 100 mg/dL. Chronic kidney disease (CKD) was defined as an estimated glomerular filtration rate (eGFR) of less than 60 mL/min/1.73 m^2^, which is equal to or more than stage III CKD.^10^ Previous MI history was defined as a history of MI before first-time PCI, accompanied by a 3-fold elevation of cardiac enzymes from the baseline value. The CAP was measured via a pigtail catheter while performing a coronary angiography. Related clinical parameters including baseline characteristics, hemodynamic data, related risk factors, and invasive strategies were compared with patients with repeated PCI and those with single PCI. In addition, we intended to identify the significant clinical factors for patients requiring repeated PCI, and identify the risk factors for MI attack, CV mortality, and all-cause mortality in patients with repeated PCI.

### Statistical Analysis

The analysis was primarily used to compare the differences between the 2 groups. Independent *t* tests, chi-square test, Fisher exact test, and multivariate logistic regression analysis were used. The log-rank test and Kaplan–Meier curves were used for the survival analysis. The Cox proportional-hazards model was used to test the effect of independent variables on hazards. *P* values less than 0.05 were considered significant. All the analyses were performed using the statistical package SPSS for Windows (Version 19.0, SPSS Inc., Chicago, IL).

## RESULTS

During the study period, a total of 1126 patients who received PCI procedure were collected. An angiographic follow-up was completed in 338 patients due to ischemia-driven clinical events and the angiographic follow-up rate was 30.0 %. Among them, 276 patients received repeated PCI procedures. Therefore, 850 patients received a single PCI procedure. The mean follow-up times for the single and repeat PCI groups were 96.8 weeks and 160.6 weeks, respectively (*P* < 0.001).

The baseline clinical characteristics were listed in Table 1. There were no significant age differences between the repeated PCI and single PCI groups (63.96 ± 10.81 vs 64.33 ± 12.33 years old). As for the body habitus parameters, compared with the single PCI group, the repeated PCI group had higher body heights (*P* = 0.016) and heavier weight (*P* = 0.019), but there were no differences in BMI. As for the hemodynamic parameters, the repeated PCI group had a higher CSP (139.87 ± 24.34 vs 135.96 ± 23.27 mm Hg; *P* = 0.017) and a higher CPP (66.69 ± 22.74 vs 62.48 ± 20.11 mm Hg; *P* = 0.006). However, there was no significant difference in CMP between the 2 groups. As for the baseline biochemistry, the repeated PCI group had a lower HDL-C level (*P* = 0.03), higher LDL-C level (*P* = 0.001), and higher cholesterol level (*P* = 0.016).

**TABLE 1:**
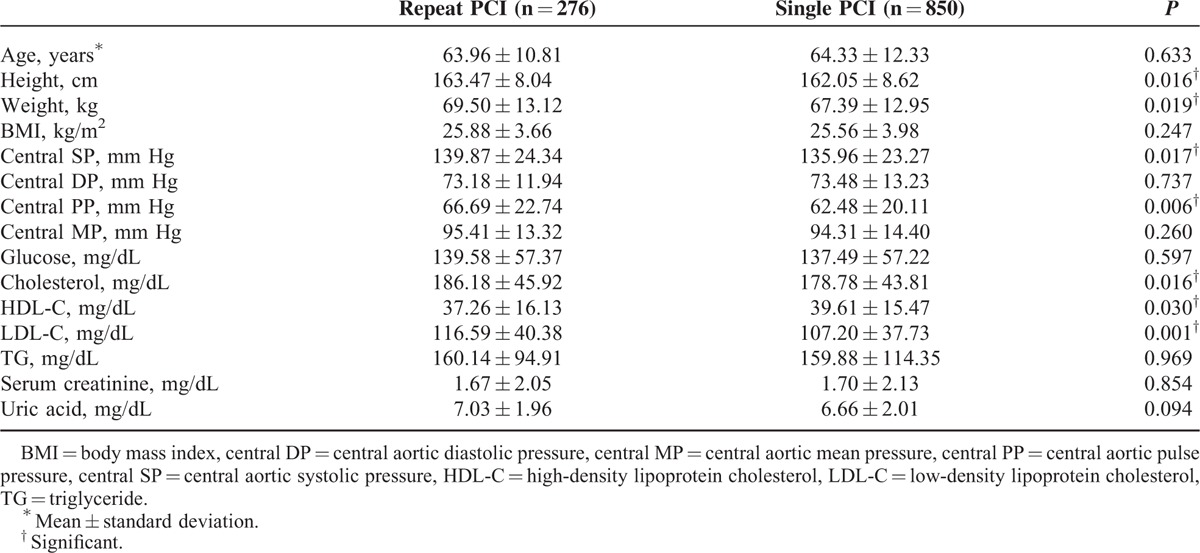
General Characteristics of the Study Population

**FIGURE 1:**
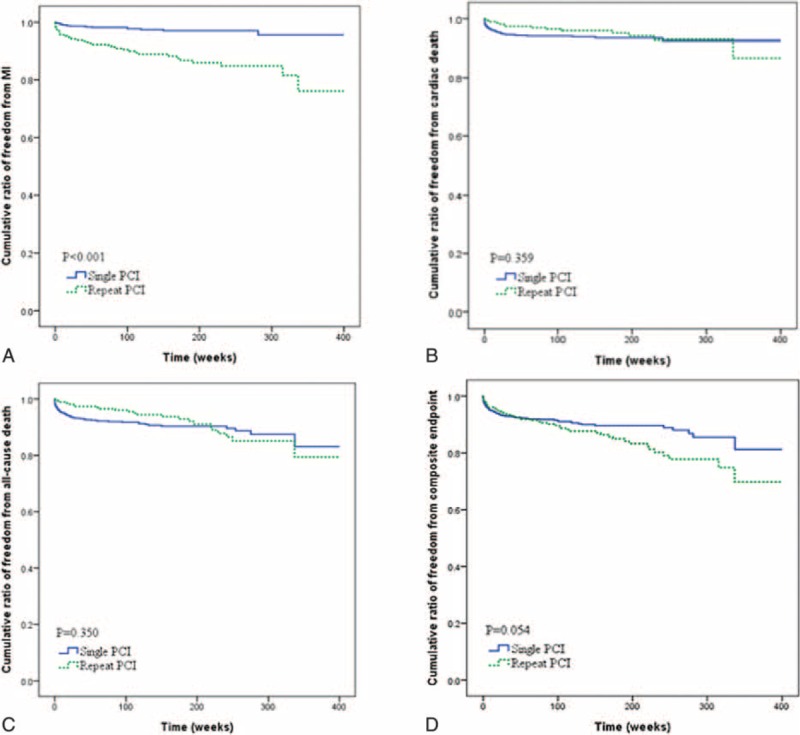
A, Cumulative ratio of freedom from myocardial infarction between 2 groups (*P* < 0.001). B, Cumulative ratio of freedom from cardiovascular mortality between 2 groups (*P* = 0.359). C, Cumulative ratio of freedom from all-cause mortality between 2 groups (*P* = 0.350). D, Cumulative ratio of freedom from composite endpoint (*P* = 0.054).

The demographic data of the study population with PCI procedure is shown in Table 2. Male participants was more prone to receive repeated PCI than their female counterparts (*P* = 0.012). In addition, we found patients in the repeated PCI group used more aspirin, BBs, and ACEIs, but less diuretics compared with those in the single PCI group (all *P* < 0.05). The results of angiographic findings and clinical outcomes are shown in Table 3. The distribution of diseased vessels in the repeated PCI group and the single PCI group was as follows: single vessel disease, 27.2% versus 53.9%; dual vessel disease, 36.2% versus 26.6%; and triple vessel disease, 36.6% versus 19.5% (*P* < 0.001). The repeated PCI group patients had a higher rate of de novo MI than the single PCI group (*P* < 0.001). Figure 1 also showed the cumulated rate of freedom from MI, CV death, all-cause death, and composite endpoint between the 2 groups. Freedom from MI was lower in the repeated PCI group than in the single PCI group (*P* < 0.001); however, there were no differences as for CV death, all-cause death, and composite endpoint between 2 groups.

**TABLE 2:**
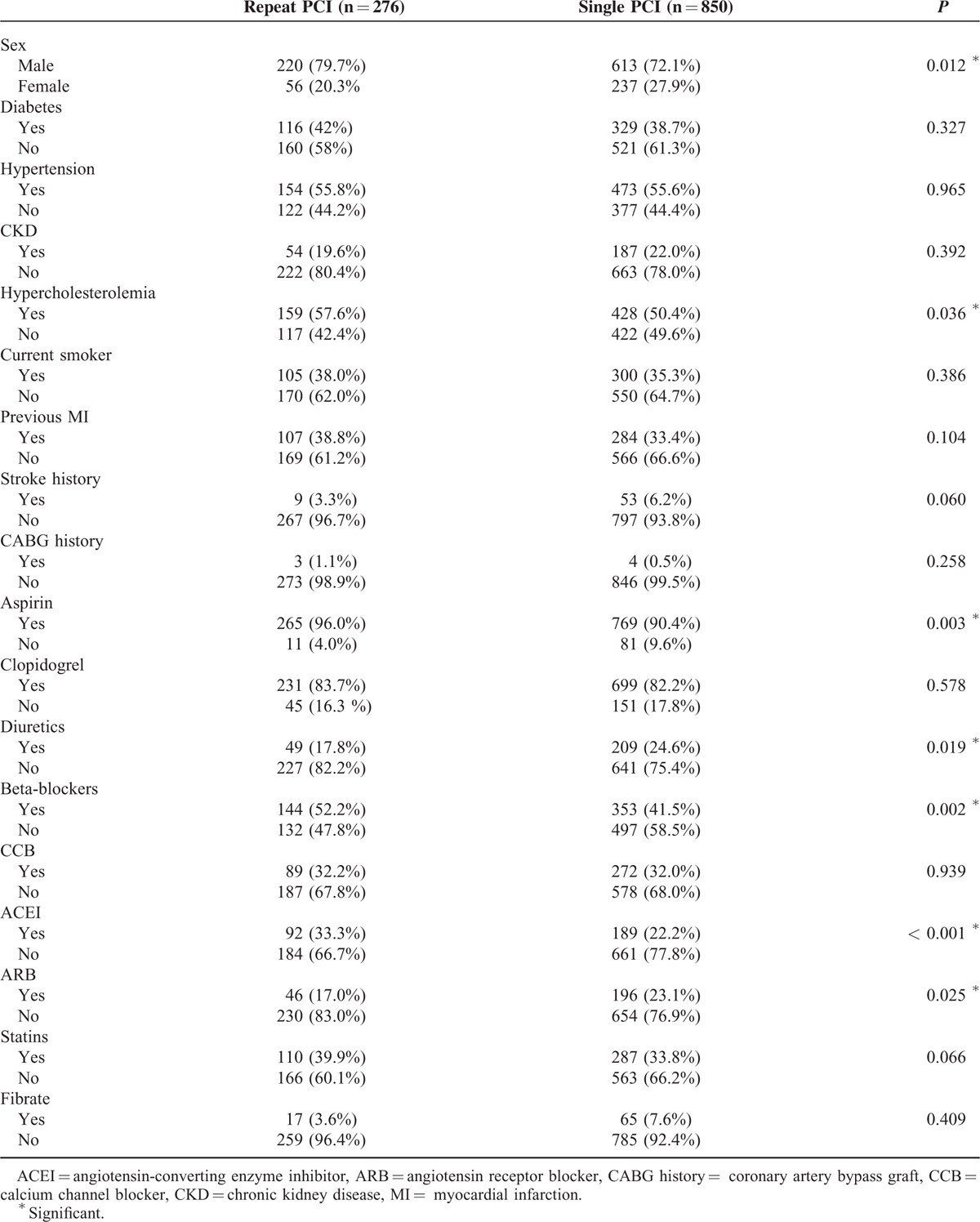
Demography of Study Population and Medications Used After First Time PCI

**TABLE 3:**
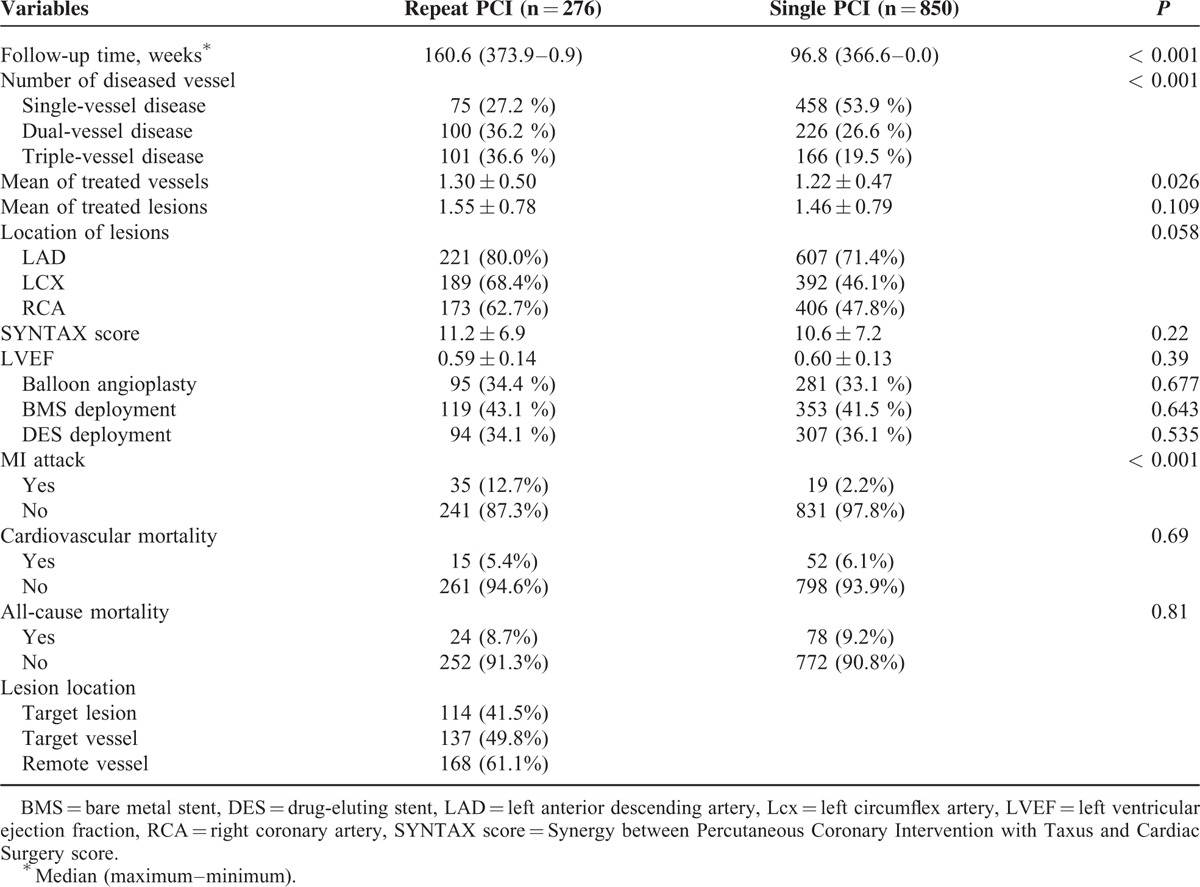
Comparison of Angiographic Findings and Outcome

The related factors in predicting the repeated PCI procedure are shown in Table 4. Based on the results of multivariate logistic regression analysis, we found that 8 independent variables, namely, male sex, CPP, usage of aspirin, usage of diuretics, usage of ACEI, usage of BB, number of diseased vessel, and presence of MI, were significant predictors for patients requiring repeated PCI procedures. Furthermore, the risk factors associated with clinical outcome in patients with repeated PCI are listed in Table 5. Previous MI history and CPP were both predictors for MI attack (*P* = 0.001, *P* = 0.019, respectively); however, only CPP was the significant risk factor for MI attack, CV mortality, and all-cause mortality in patients with repeated PCI (*P* = 0.019, *P* = 0.041, *P* = 0.004, respectively). In model 2, we selected CSP to replace CPP as a parameter for multivariate logistic regression analysis, and found CSP was also shown as a significant predictor for patients requiring repeated PCI procedures (*P* = 0.004). In addition, based on the results of Cox proportional-hazards model 2, previous MI was still a predictor for MI attack and all-cause mortality, but CSP was, however, only the significant risk factor for all-cause mortality (*P* = 0.018), and not for MI attack (*P* = 0.123) and CV mortality (*P* = 0.091).

**TABLE 4:**
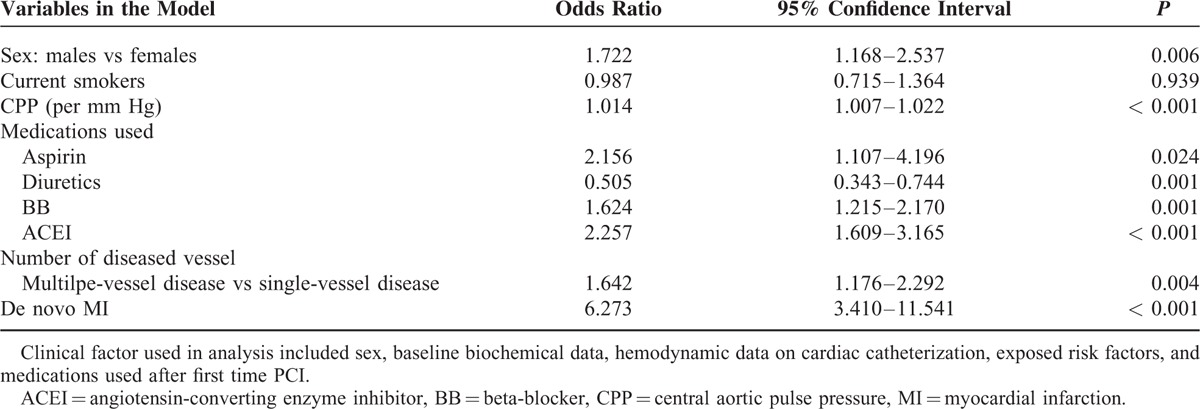
Significant Predictors of Repeat PCI in Stepwise Multiple Logistic Regression

**TABLE 5:**
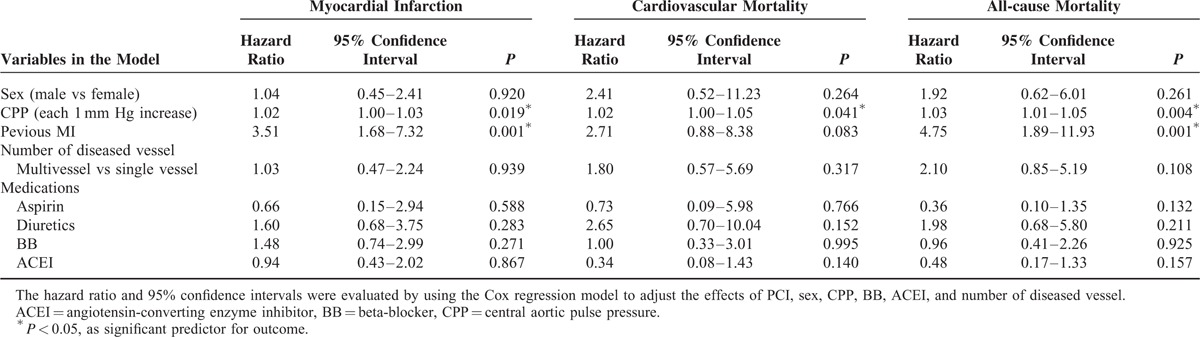
Significant Predictors of Outcome in Cox Proportional-hazards Model in Repeat PCI Group

## DISCUSSION

Patients with ischemic heart diseases represent a population exposed to a high risk of cardiac events when treated medically. Despite relevant progress in treatment, prognosis in this population remains poor. Although some relatively new methods such as mesenchymal stem cells could exhibit protective effects against many kinds of diseases including MI, the clinical application of the methods requires further investigation.^12–14^ As we know, repeated PCI is still necessary and indicated for patients with CAD. In this study, elevated CPP, higher rate of de novo MI, male sex preponderance, and more usage of diuretics, BB, and ACEI in patients with repeated PCI were found to be significant parameters in patients with repeated PCI compared with those with single PCI. Therefore, clinically, male sex, elevated central PP, usage of diuretics, usage of ACE inhibitors, usage of BB, number of diseased vessels, and de novo MI could be potential risk factors associated with patients receiving repeated PCI.

In our study, we found a higher CPP was a strong factor for patients to receive repeated PCI. As we know, elevated CPP was an important factor in some previous studies^11^; they reported that the CPP was 3 mm Hg lower in patients who received amlodipine plus perindopril than in patients who received thiazide plus atenolol, and the elevated CPP was related to the post hoc defined composite outcome of total CV event/procedures and the development of renal dysfunction. However, the CPP was calculated via indirect measurement through radial artery applanation tonometry and pulse wave analysis in the Conduit Artery Function Evaluation (CAFE) study, whereas in our study, the CPP was measured via a pigtail catheter, and this may have more exactly reflected the hemodynamic difference and vessel wall stress between the 2 groups. Some studies also reported the efficacy of reducing CPP was a reason to explain the better outcome in the group of patients who received amlodipine plus perindopril.^15^ In another study for patients with end-stage renal disease, CPP had a more predictive value for a major adverse CV event than for brachial artery pressure.^16^ Increased fractional pulse pressure (ratio of pulse pressure to mean pressure) has been reported to be a predictor for restenosis after PCI in 1 previous study.^17^ Therefore, we think it is an important issue to control CPP appropriately to reduce the possibility of repeated PCI and gain a better outcome in patients with repeated PCI.

In addition, aspirin, ACEIs, and BBs were all used more frequently in the repeated PCI group than in the single PCI group in our study. We found in aspirin users, repeated PCI group had significantly higher comorbidities such as dyslipidemia and previous history of MI than single PCI group. In patients receiving ACEIs, the repeated PCI group had more advancing age and current smokers than the single PCI group, which means they had more complex clinical conditions. On the other hand, for those patients who used BB, the comorbidity was not different between the 2 groups. BBs indeed have a less protective effect in patients with CAD.^18,19^ Moreover, the number of diseased vessels was found to have a positive correlation with repeated PCI procedures. Triple vessel disease seemed to be the most common diseased vessel numbers in patients with repeated PCI, followed by dual diseased vessels, whereas single diseased vessel was more common in patients with single RCI. Clinically, primary care clinicians should pay attention to the number of diseased vessels. Once the number of diseased vessels increases, the possibility of repeated PCI will increase. Although location and complexity of the lesions did not show statistically significant difference between patients with repeated PCI and those with single PCI, we still found LAD was the most common location of lesions in patients with both single and repeated PCI. However, Lcx was the second common location of lesions in patients with repeated PCI, whereas RCA was the second common in patients with single PCI.

Furthermore, we analyzed the risk factors for clinical outcomes as MI attack and mortality in patients repeated PCI. In our study, we noted that patients with repeated PCI suffered from higher rates of de novo MI attack. On the basis of the results of Cox proportional-hazards model, CPP and previous history of MI were considered as significant factors for patients with MI attack after performing repeated PCI, and CPP seemed to be a significant risk factor for both CV mortality and all-cause mortality in our study. Clinically, CPP could be a very important parameter for clinical outcome, not only MI attack but also mortality, in patients with repeated PCI. Carefully monitoring and controlling CPP should be advised in patients with repeated PCI to achieve a better prognosis. In addition, patients with a history of previous MI should also be mentioned in the history taking and recorded in the medical chart, because this history was also a risk factor for re-attack of MI in patients with repeated PCI.

Nevertheless, there were some limitations in this study. First, given geographical and country differences in clinical practice, the difference of patient selection may exist. Second, plaque composition analysis and fraction flow reserve measurement were not used in our angiographic study, which may also had an impact on index PCI. Third, mechanisms for repeat PCI such as instent restenosis, stent thrombosis, or de novo atherosclerosis were not further analyzed. Finally, since this is a prospective cohort study, whether reduced CPP could improve outcome in patients after receiving index PCI remains to be clarified by large randomized clinical trials.

In conclusion, elevated CPP, CSP, male sex, multiple diseased vessels, and the usage of diuretics, BBs, ACEIs, and MI were predictors for repeated PCI. Most importantly, CPP was strongly associated with MI attack, CV mortality, and all-cause mortality, and could serve as a prognostic parameter for mortality in patients with CAD after performing repeated PCI.
