# Prognostic Significance of Central Pulse Pressure for Mortality in Patients With Coronary Artery Disease Receiving Repeated Percutaneous Coronary Intervention: Erratum

**DOI:** 10.1097/01.md.0000482813.61604.2c

**Published:** 2016-05-06

In the article “Prognostic Significance of Central Pulse Pressure for Mortality in Patients With Coronary Artery Disease Receiving Repeated Percutaneous Coronary Intervention”,^1^ which appeared in Volume 95, Issue 13 of *Medicine*, the authors’ country was originally given incorrectly. The article has since been corrected online.
